# (Digitale) Elternzusammenarbeit in Kindertageseinrichtungen während der Corona-Pandemie. Digitalisierungsschub oder verpasste Chance?

**DOI:** 10.1007/s11618-021-01014-7

**Published:** 2021-04-16

**Authors:** Franziska Cohen, Elisa Oppermann, Yvonne Anders

**Affiliations:** 1 Institut für Erziehungswissenschaft – Abteilung Kindheitspädagogik, Pädagogische Hochschule Freiburg, Kunzenweg 21, 79117 Freiburg, Deutschland; 2 Lehrstuhl für Frühkindliche Bildung und Erziehung, Otto-Friedrich-Universität Bamberg, Markusstraße 8a, 96045 Bamberg, Deutschland

**Keywords:** Elternzusammenarbeit, Digitalisierung, IKT, Corona, Kindertageseinrichtung, Corona, Day-care cetre, Digitalization, ICT, Parent-educator-cooperation

## Abstract

Die Schließung von Kindertageseinrichtungen (Kita) als Maßnahme zur Eindämmung des Corona-Virus stellte frühpädagogische Fachkräfte kurzfristig vor veränderte Tätigkeitsbedingungen und Möglichkeiten, den weiterhin bestehenden Bildungsauftrag umzusetzen. Die Zusammenarbeit mit Eltern, mit und ohne digitale Medien, spielt in dieser Hinsicht eine wichtige Rolle. Der Beitrag untersucht a) wie häufig und in welcher Form Fachkräfte die Elternzusammenarbeit in der Corona-Schließzeit umsetzten, b) welche Einstellungen frühpädagogische Fachkräfte zu digital-gestützter und allgemeiner Elternzusammenarbeit in der Corona-Schließzeit hatten und c) welche Rolle die Qualifikation der Fachkräfte, ihre Einstellungen und ihre wahrgenommene Unterstützung im Hinblick für die digitale und nicht digitale Elternzusammenarbeit in der Corona-Schließzeit spielten. Datenbasis bildet eine bundesweite Onlinebefragung von 3513 Fachkräften in Kitas während der Corona-Schließzeit. Die Ergebnisse zeigen, dass die wahrgenommene eigene Rolle im Hinblick auf die Elternzusammenarbeit einen positiven Einfluss darauf hat, ob mit den Eltern Kontakt aufgenommen wurde. Ob dieser Kontakt über digitale Medien passiert, hängt unter anderem von den Einstellungen der Fachkräfte zu digitalen Medien, der erwarteten Reaktion der Eltern auf diese Form der Elternzusammenarbeit und von der technischen Unterstützung im Implementationsprozess digitaler Medien in der Elternzusammenarbeit ab. Die Ergebnisse werden in Bezug auf strukturelle Bedingungen von Einrichtungen und professionelle Kompetenzen frühpädagogischer Fachkräfte für eine breite Implementierung digitaler Medien in der frühpädagogischen Praxis diskutiert.

## Abstract (en)

The closure of day-care centres as a measure to contain the corona virus presented early childhood educators with changed working conditions and opportunities to implement the continuing educational mandate. The cooperation between parents and educators, including through digital tools, plays an important role in this respect. The paper examines a) how often and of what type early childhood educators implemented digitally supported parental cooperation during the closure of day-care centres, b) which attitudes educators had towards general as well as digitally supported parental cooperation, and c) what role their qualifications, their attitudes and their perceived support through the centre played for their digital and non-digital parental cooperation during the closure of day-care centres. The study is based on a nationwide online survey of 3513 early childhood educators in day-care centres during corona closing time. The results show that educators’ perceived importance of parental cooperation had a positive influence on whether or not educators had stayed in contact with the parents. Whether this contact occurs via digital media, in turn, depended on educators’ attitudes towards digital media, the expected reaction of parents to this form of parental cooperation and the availability of technical support for implementing digital media in parental cooperation. The results are discussed with regard to the structural conditions of institutions and the professional skills of early childhood educators for a broad implementation of digital media in early childhood education practice.

## Einleitung

Für Kinder sind neben der Familie die Einrichtungen zur frühkindlichen Bildung, Betreuung und Erziehung ein wichtiges Entwicklungs- und Lernumfeld (Bronfenbrenner und Morris 2006). Die Bedeutsamkeit qualitativ hochwertiger pädagogischer Praxis in Kindertageseinrichtungen (Kita) auf die soziale und kognitive Entwicklung von Kindern ist gut belegt (Anders 2013; Ulferts et al. 2019). Die Zusammenarbeit mit Familien nimmt hierbei eine zentrale Rolle ein, da sie beide Lern- und Entwicklungsumwelten verbindet und somit Kinder in ihrer Entwicklung und ihrem Wohlbefinden unterstützen kann (Lehrl et al. 2020). Eltern und Einrichtungen agieren damit in gemeinsamer Verantwortung für das kindliche Wohl und die kindliche Entwicklung (§ 22 a SGB VIII Abs. 2).

Die Schließung der Kitas als Maßnahme zur Eindämmung des Corona-Virus im März 2020 stellte die Kitas sehr kurzfristig vor eine veränderte Situation, die die direkte Arbeit mit Kindern und Eltern unmöglich machte. Gleichzeitig bestand – wie auch für Schulen – weiterhin der Bildungsauftrag, wie er durch die Jugend- und Kultusministerkonferenz im Gemeinsamen Rahmen der Länder für die frühe Bildung festgelegt und in den Bildungs- und Orientierungsplänen der Länder präzisiert ist. Der Bildungsauftrag von Kitas umfasst dabei die ganzheitliche Stärkung frühkindlicher Kompetenzen und wird sowohl über die unmittelbare Arbeit am Kind als auch die Zusammenarbeit mit den Eltern im pädagogischen Alltag umgesetzt (Jugend- und Kultusministerkonferenz 2004).

Der Einsatz von Informations- und Kommunikationstechnologien (IKT)^1^, sowohl in der kindbezogenen pädagogischen Arbeit als auch in der Zusammenarbeit mit Eltern, spielte in Kitas bisher eine untergeordnete Rolle (Autorengruppe Bildungsberichterstattung 2020; Knauf 2020b; Viernickel et al. 2013), wurde aber im Hinblick auf die Aufrechterhaltung des Kontakts zwischen den Eltern und frühpädagogischen Fachkräften in der Corona-Schließzeit plötzlich relevanter. Der vorliegende Beitrag setzt hier an und untersucht die Zusammenarbeit zwischen Fachkräften und Eltern, sowie die Bedingungen für eine digital-unterstützte Elternzusammenarbeit während der Corona-Schließzeit.

IKT bieten für die Fachkräfte eine erweiterte Möglichkeit, die Zusammenarbeit mit den Familien auch ohne den persönlichen Kontakt zu den Eltern umzusetzen und darüber dem Bildungsauftrag nachzukommen. Die Pandemie und ihre Folgen könnten damit einen Digitalisierungsschub in Kitas ausgelöst haben. Ein Digitalisierungsschub würde sich damit sowohl auf die rein technische Ausstattung der Einrichtungen als auch auf die Nutzung dieser für pädagogische Ziele in der Arbeit mit Kindern und Familien beziehen. Bisher gibt es nur wenige Untersuchungen zur Umsetzung digitalgestützter Elternzusammenarbeit. Dennoch zeigen die kontroversen Debatten um die Relevanz von IKT in der praktischen Bildungsarbeit, wie divers Einstellungen von Fachkräften und Wissenschaftlern auch grundsätzlich zum Thema IKT in Kitas diskutiert werden (Fröhlich-Gildhoff und Fröhlich-Gildhoff 2017; Institut für Demoskopie Allensbach 2014).

Im Folgenden wird zunächst die IKT-gestützte Elternzusammenarbeit in ihrer bisherigen Umsetzung sowie bezüglich ihrer Vor- und Nachteile beleuchtet. Daran anschließend wird die bisherige empirische Evidenz wichtiger Prädiktoren für den Einsatz von IKT in der Elternzusammenarbeit zusammengefasst und das analyseleitende theoretische Modell vorgestellt, um darauf aufbauend die Fragestellungen zu präsentieren. Die methodische Umsetzung der Studie, sowie die Ergebnisse und Diskussion der Studie folgen.

## (Digitale) Elternzusammenarbeit in Einrichtungen zur frühkindlichen Bildung, Betreuung und Erziehung

Ziel von Elternzusammenarbeit ist es, eine vertrauensvolle positive Beziehung aufzubauen, die den Grundstein für einen gegenseitigen Austausch sowie die gelungene Mitbestimmung und Beteiligung von Eltern legt (Viernickel et al. 2013). Damit soll eine Entkopplung von Familie und Institution hinsichtlich der Erziehung und Bildung vermieden werden und vielmehr die Erziehungskompetenz der Eltern gestärkt werden, statt die Erziehungsverantwortung an die frühpädagogischen Fachkräfte zu übertragen (Olmstead 2013; Swick 2003; Textor and Blank 2004). Die Zusammenarbeit zwischen Eltern und Fachkräften in Kitas nimmt damit einen wichtigen Stellenwert in der pädagogischen Arbeit ein, der durch die konzeptionelle Verankerung in den Bildungsplänen unterstrichen wird (Betz 2015).

Mit dem Hinweis, dass in der Debatte um die Einbeziehung der Eltern die Begriffe Elternarbeit und Elternpartnerschaft kritisiert wurden, wird im vorliegenden Beitrag mit Bezug auf die Definition von Betz (2015) unter Elternzusammenarbeit jegliche Form der organisierten Kommunikation und Kooperation zwischen frühpädagogischen Fachkräften und Eltern verstanden. Mit dem Begriff Elternzusammenarbeit wird dabei der kooperative Charakter der Beziehung unterstrichen (Fröhlich-Gildhoff 2013).

Eine Reihe von Studien zeigt, dass eine gute Zusammenarbeit und Kommunikation zwischen Eltern und Fachkräften mit einer höheren Zufriedenheit der Eltern mit der Einrichtung (Elicker et al. 1997; Nzinga-Johnson et al. 2009) sowie einer positiven Entwicklung kognitiver und sozial-emotionaler Kompetenzen von Kindern einhergeht (Fan und Chen 2001; Lehrl et al. 2020; Van Voorhis et al. 2013; Wilder 2014). Gängige Formen der Elternzusammenarbeit in Kitas sind Elternabende oder gemeinsame Feste, Tür- und Angelgespräche und Entwicklungsgespräche. Weniger verbreitet sind zum Beispiel Hausbesuche und die Beteiligung der Eltern an der Konzeptentwicklung (Fröhlich-Gildhoff 2013; Gragert et al. 2008; Viernickel et al. 2013).

Die zunehmende Verbreitung und Weiterentwicklung digitaler Technologien bietet neue Möglichkeiten zur Unterstützung der Elternzusammenarbeit (Hall und Bierman 2015). IKT sind niedrigschwellig und die Kommunikation ist asynchron. Somit könnten idealerweise auch Eltern für die gemeinsame Arbeit gewonnen werden, die bisher als schwer erreichbar galten. IKT werden sowohl von Eltern als auch Fachkräften als effizienter, flexibler, unmittelbarer, effektiver und bequemer als traditionelle Formen der Zusammenarbeit eingeschätzt (Ho et al. 2013; McFadden und Thomas 2016; Olmstead 2013; Snell et al. 2018; Yost und Fan 2014), was wiederum die Engagiertheit der Eltern und deren Zufriedenheit mit der Elternzusammenarbeit stärkt (Olmstead 2013; Wasserman und Zwebner 2017).

Bedenken zeigen sich eher in Bezug auf ethische Aspekte und die Art der Inhalte (McFadden und Thomas 2016; Yost und Fan 2014). Dabei ist es vor allem entscheidend, dass die Kommunikation über IKT nicht den persönlichen Austausch ersetzt (Knauf 2020b; McFadden und Thomas 2016; Wasserman und Zwebner 2017; Yost und Fan 2014). Wasserman und Zwebner (2017) weisen darauf hin, dass der stärkere Zugang der Eltern zum Handlungsfeld der Fachkräfte etwas Neues ist, was durch die digitale Kommunikation intensiviert wurde. Das passiert vor allem durch den vermehrten Kontakt und die Tatsache, dass digitale Informationen sowohl Fachkräfte als auch Eltern überall, jederzeit und ununterbrochen begleiten können. Wasserman und Zwebner (2017) sehen darin auch Herausforderungen. Die Grenze zwischen Institution und Familie verschwimmt, was wiederum dazu führen könnte, dass eine Illusion von Freundschaft zwischen Fachkräften und Eltern entsteht oder dass Eltern übermäßig stark involviert werden möchten (Kurtz 2014). Dies ist auch gegeben, wenn Fachkräfte unprofessionell kommunizieren (z. B. Duzen, private Themen besprechen). Damit einher geht ebenfalls eine geringere Hemmschwelle, private Informationen in die Öffentlichkeit zu senden (in diesem Fall die Einrichtung). Ebenfalls geben Wasserman und Zwebner (2017) zu bedenken, dass die häufige und unmittelbare Kommunikation von den beteiligten Partnern eine hohe Kompetenz im Umgang mit der Interpretation von Nachrichten, der Filterung von Inhalten und der Regulation der Informationsflut abverlangt (Flynn und Nolan 2008; Shechtman und Boucherian 2015; Wasserman und Zwebner 2017). Im Zusammenhang mit digitaler Bildungsdokumentation wird ebenfalls diskutiert, dass Eltern als Adressaten stärker in den Mittelpunkt rücken, entsprechend Informationen anders aufbereitet werden und sich damit eher „zum Schaufenster für die pädagogische Arbeit“ entwickeln (Knauf 2020a).

Im Zuge der Schließungen der Kitas gewann die digitale Zusammenarbeit mit Familien schlagartig an Bedeutung, da sie die einfachste Möglichkeit für Fachkräfte war, ihren Bildungsauftrag umzusetzen. Die Integration von IKT in die Elternzusammenarbeit war bis dato jedoch nicht flächendeckend gelungen (Knauf 2020b; Viernickel et al. 2013). Bekannte IKT in der Elternzusammenarbeit sind E‑Mails, Messenger-Programme oder geschützte webbasierte Plattformen zum Austausch von Informationen zwischen Eltern und Fachkräften und zur digitalen Dokumentation (Burghardt und Knauf 2015; McFadden und Thomas 2016). Eine Studie von Knauf (2020b) mit 190 frühpädagogischen Fachkräften zeigte, dass analoge, papierbasierte Formen der Kommunikation, im Vergleich zu digitalen Formen, jedoch deutlich überwiegen. E‑Mails wurden immerhin von einem Viertel der Einrichtungen häufig oder manchmal genutzt, Messenger oder Online-Portale dagegen nur sehr selten (Knauf 2020b). Auch Viernickel et al. (2013) stellten bereits 7 Jahre früher fest, dass nur etwa 11 % der Fachkräfte E‑Mailverteiler nutzen.

Welche Voraussetzungen für den Einsatz von IKT im Kontext frühkindlicher Bildungsinstitutionen notwendig sind, soll im folgenden Kapitel erörtert werden.

## Theoretisches Modell zur digitalen Elternzusammenarbeit

Um einen Rahmen zur Einordnung der Elternzusammenarbeit in die pädagogische Qualität von Kindertageseinrichtungen zu setzen, wird das strukturell-prozessuale-Modell pädagogischer Anregungsqualität als ein mehrdimensionales Konstrukt mit den vier Hauptdimensionen Strukturqualität, Orientierungsqualität, Prozessqualität und die Vernetzung mit den Familien beschrieben (Kluczniok und Roßbach 2014). Die strukturelle Dimension umfasst Merkmale, die die gegebenen Rahmenbedingungen für die pädagogische Arbeit repräsentieren, z. B. der Fachkraft-Kind-Schlüssel. Merkmale der Orientierungsqualität beziehen sich auf pädagogische Einstellungen, wie z. B. das pädagogische Selbstverständnis, Überzeugungen darüber, wie Kinder lernen, oder Einstellungen zur Elternzusammenarbeit. Dem liegt die Annahme zugrunde, dass Einstellungen richtungsleitend sind und eine Entscheidungsgrundlage für die eigene pädagogische Praxis darstellen (Anders 2012). Die Prozessqualität wird über die Quantität und Qualität von Interaktionen zwischen Kindern, Kindern und Fachkräften sowie das Angebot an bildungsrelevanten Aktivitäten beschrieben (Kluczniok und Roßbach 2014). Eine vierte Dimension des Modells ist die „Vernetzung mit den Familien“, welche Aspekte der Elternzusammenarbeit abdeckt (Kluczniok und Roßbach 2014). Obwohl Elternzusammenarbeit bereits seit langem ein zentrales Thema pädagogischer Arbeit in Kitas ist, gibt es bisher nur wenige Untersuchungen, in denen diese Dimension zur Elternzusammenarbeit in ihren Zusammenhängen mit den Struktur- und Orientierungsmerkmalen untersucht wurde (Anders et al. 2016^2^; Murray et al. 2015).

Ebenfalls weist das strukturell-prozessuale Modell pädagogischer Qualität Limitierungen auf. So werden die Merkmale der einzelnen Fachkraft (z. B. ihre Qualifikation) nicht konkret berücksichtigt. Ebenfalls ist unklar, ob sich Zusammenhangsstrukturen des Modells bzgl. der Prozessqualität auf die Dimension Zusammenarbeit mit Familien übertragen lassen. So wird bspw. angenommen, dass Strukturen und Orientierungen wichtige Voraussetzungen für die Handlungspraxis in verschiedenen Bildungsbereichen darstellen (Kluczniok et al. 2011; Slot 2018; Kratzmann et al. 2020). Für die Elternzusammenarbeit, sowohl digital als auch nicht digital, wurde dies bisher nicht hinreichend empirisch überprüft.

Die Bedeutsamkeit der Rahmenbedingungen sowie der Einstellungen für die Handlung findet sich auch in Modellen zur IKT-gestützten Handlungspraxis im bildungswissenschaftlichen Kontext wieder. Grundsätzlich werden hier Barrieren in den Strukturen und Orientierungen getrennt beschrieben (Blackwell et al. 2014): Als Barrieren erster Ordnung werden die externen Rahmenbedingungen, z. B. die technische Ausstattung, der Zugang dazu und die technische Unterstützung definiert. Die Barrieren 2. Ordnung umfassen die Einstellungen von Fachkräften im Hinblick auf die Anwendung digitaler Medien im Bildungskontext. In erweiterten theoretischen Modellen zur Nutzung und Akzeptanz von IKT, z. B. „Will, Skill, Tool Model“ (Knezek et al. 2003; Knezek und Christensen 2016) oder „Technology-Acceptance Model“ ((Davis et al. 1989), bzw. „UTAUT“ (Venkatesh und Davis 2003)), wird die Bedeutung von Einstellungen gegenüber der Technologie für das Handeln einer Person bzw. die Akzeptanz und den Einsatz digitaler Medien ebenfalls hervorgehoben und um motivationale und infrastrukturelle Aspekte ergänzt. Diese Modelle erweitern somit die Grundannahme der Relevanz von Strukturen und Überzeugungen für den IKT-Einsatz um externe Faktoren, wie zum Beispiel der wahrgenommenen Unterstützung oder dem Teamklima.

**Figure 1:**
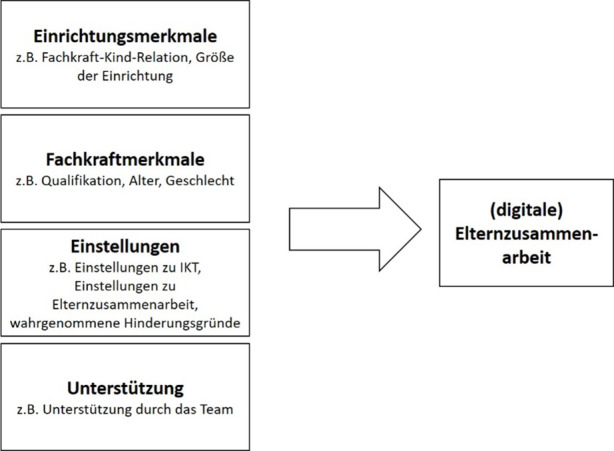

Der vorliegenden Arbeit wird ein analyseleitendes Modell zugrunde gelegt (Abb. 1), welches sowohl die Einstellungen als auch die strukturellen Aspekte als Prädiktoren für die Implementierung von IKT in der Zusammenarbeit mit Eltern während der Corona-Schließzeit betrachtet. Dabei umfassen die strukturellen Merkmale Informationen zur Einrichtung (z. B. Einrichtungsgröße) und zu den Fachkräften (z. B. Qualifikation). In Bezug auf die Überzeugungen sind sowohl Einstellungen zur Elternzusammenarbeit als auch zu IKT relevant. Einstellungen sind als Teil der professionellen Haltung und als Facette professioneller Kompetenzen zu definieren (Anders 2012; Fröhlich-Gildhoff et al. 2011). Sie bilden Vorstellungen, Haltungen und Werte von Fachkräften ab, wie z. B. das Rollenverständnis in Bezug auf den Bildungsauftrag. Einstellungen umfassen ebenfalls motivationale und emotionale Aspekte des professionellen Handelns (z. B. Interesse, Ängste). Somit können auch die von den Fachkräften antizipierten Hinderungsgründe in der Umsetzung als Einstellungsmerkmale gefasst werden (Plumb und Kautz 2015). Erweitert wird das Modell durch Aspekte der Unterstützung in der Implementierung.

Zusammenhänge zwischen der (digitalen) Elternzusammenarbeit und ihren Prädiktoren wurden bereits in verschiedenen, überwiegend internationalen Studien untersucht. Das folgende Kapitel fasst die Ergebnisse zusammen.

## Forschungsergebnisse zu Einflussfaktoren auf digitale Elternzusammenarbeit.

Während es nur wenige Studien gibt, die die Zusammenarbeit von Eltern und Fachkräften und ihre Prädiktoren betrachten, sind Untersuchungen zur IKT-gestützten Elternzusammenarbeit im Kontext der frühkindlichen Bildung im internationalen Kontext selten und im deutschsprachigen Raum gar nicht vorhanden. Im Folgenden werden deshalb auch Studien berichtet, die sich auf die Nutzung von IKT in der frühpädagogischen Praxis in Bildungssituationen und auf die nicht digitale Elternzusammenarbeit beziehen. Auch wenn die Übertragbarkeit von Studien limitiert ist, lohnt es sich, beide empirischen Stränge zu verknüpfen, um sich dem Untersuchungsthema zumindest anzunähern.

Mit Blick auf die traditionellen Formen der Elternzusammenarbeit zeigen Castro et al. (2004) und Anders (2016^3^), dass *Fachkraftmerkmale* wie die Berufserfahrung und die Qualifikation wichtige Prädiktoren für die Intensität der elterlichen Beteiligung in der Zusammenarbeit sind. In anderen Studien wiederum konnte dieser Zusammenhang nicht nachgewiesen werden (Perlman und Fletcher 2012; Swartz und Easterbrooks 2014). In Bezug auf die Nutzung digitaler Medien in Bildungssituationen konnten Bastian et al. (2018) und Marci-Boehncke et al. (2012) einen positiven Effekt von Qualifizierungsmaßnahmen, insbesondere auf die Einstellungen zur Nutzung digitaler Medien feststellen.

Ebenfalls belegen verschiedene nationale und internationale Studien die Relevanz positiver *Einstellungen* gegenüber der Nutzung von IKT in Bildungssituationen. Sie sind vor allem für die Art und Weise, wie digitale Medien im pädagogischen Alltag eingesetzt werden, prädiktiv (Blackwell et al. 2014; Kerckaert et al. 2015; Lindahl und Folkesson 2012; Plumb und Kautz 2015). Gleichzeitig wird der Einsatz von IKT in Kitas im politischen und wissenschaftlichen Diskurs kontrovers diskutiert (Fröhlich-Gildhoff und Fröhlich-Gildhoff 2017). Diese skeptische bis negative Haltung gegenüber dem Einsatz von IKT lässt sich auch bei vielen Fachkräften in der Praxis belegen, wobei sich die Befunde jedoch heterogen darstellen (Brüggemann et al. 2013; Friedrichs-Liesenkötter 2019; Institut für Demoskopie Allensbach 2014; Knauf 2020b). Ein Teil der Fachkräfte scheint einer frühen Medienerziehung ablehnend gegenüberzustehen – und das unabhängig vom Alter und der Berufserfahrung (Brüggemann et al. 2013; Friedrichs-Liesenkötter 2016a). Diese skeptische Haltung zeigt sich auch bei den Eltern. Bezogen auf die digital gestützte Elternkommunikation zeigen die Ergebnisse der qualitativen Studie von Bordalba und Bochaca (2019), dass für die Einstellungen der Fachkräfte und Eltern sowohl nach genutzter Technologie selbst als auch nach dem Kontext, in dem IKT eingesetzt werden, unterschieden werden muss. Ebenfalls beschreiben die Autoren, dass die durch die Fachkraft antizipierte Einstellung anderer Personen, z. B. der Leitung oder der Eltern, bezüglich der eigenen IKT-Integration eine Rolle spielt. Für die Nutzung digitaler Medien in der pädagogischen Praxis mit Kindern nennen Fachkräfte weiterhin eine Reihe von Hinderungsgründen, die sie wahrnehmen, denen sie sich stellen und die sie ggf. überwinden müssen. Fachkräfte berichten unter anderem von einer unzureichenden technischen Ausstattung (Plumb und Kautz 2015; Schubert et al. 2018; Six et al. 2007), den verschiedenen Kommunikationsbedürfnissen und Kommunikationspräferenzen der Eltern (Yost und Fan 2014), datenschutzrechtlichen Bedenken (Eder und Roboom 2018; Yost und Fan 2014) und fehlenden zeitlichen Ressourcen (Marklund 2019; Plumb und Kautz 2015; Six et al. 2007).

Als eine weitere Voraussetzung für den generellen Einsatz von IKT in der Kita geben Fachkräfte die* Unterstützung *durch Kolleg*innen, Leitung, Träger und Eltern an (Blackwell et al. 2014; Nikolopoulou und Gialamas 2015; Six et al. 2007). Insbesondere den Leitungskräften kommt hier eine zentrale Rolle zu, um die Kommunikation mit den Familien und das Engagement der Fachkräfte und Eltern in der digitalen Elternzusammenarbeit zu stärken (Bordalba und Bochaca 2019). Dabei geht es sowohl um die inhaltliche Unterstützung des Implementationsprozesses im Hinblick auf eine fachliche und didaktische Expertise (Schubert et al. 2018) als auch um die technische Unterstützung beim Einsatz und der Instandhaltung von IKT (Bastian et al. 2018; Blackwell et al. 2014).

Der bisherige nationale und internationale Forschungsstand zeigt, dass Qualifikationsmerkmale der Fachkraft, die Einstellungen zu IKT, persönlich wahrgenommene Hinderungsgründe und die Unterstützung durch weitere Personen für die Akzeptanz und den tatsächlichen Einsatz digitaler Technologien wichtig sind. Jedoch basieren die meisten Studienergebnisse auf Untersuchungen zu traditionellen Formen der Elternzusammenarbeit und dem Einsatz von IKT in der direkten Arbeit mit Kindern. Eine Übertragung der Zusammenhänge ist problematisch, da angenommen werden kann, dass sich das Nutzungsverhalten, die Funktionen, die Einstellungen und Erfahrungen mit IKT als Kommunikationswerkzeug bei den Fachkräften anders darstellen als z. B. im Rahmen von Bildungsaktivitäten. International gibt es nicht genügend und für den deutschsprachigen Raum bisher keine empirischen Studien zu den Voraussetzungen des Einsatzes von IKT in der Zusammenarbeit zwischen frühpädagogischen Fachkräften und Eltern. Insbesondere während der coronabedingten Schließung der Kitas wurde aber deutlich, wie relevant das Wissen um die Voraussetzungen im Hinblick auf eine gelungene Implementation in der Praxis ist. Diese Forschungslücke soll mit der vorliegenden Studie Berücksichtigung finden.

## Fragestellungen

Vor dem Hintergrund des theoretischen Modells und der bisherigen empirischen Studien zu den Einflussfaktoren der Elternzusammenarbeit sowie der Nutzung von IKT im Kontext frühkindlicher Bildungseinrichtungen, lassen sich folgende Fragestellungen ableiten:

- Wie häufig und in welcher Form gestalten Leitungskräfte sowie Erzieher*innen die Elternzusammenarbeit in der Corona-Schließzeit unter besonderer Berücksichtigung von IKT?
  H1: Es wird vermutet, dass sich Leitungskräfte und Erzieher*innen bzgl. der Form und Häufigkeit des Kontaktes zu Eltern signifikant unterscheiden.
- Welche Einstellungen haben Leitungskräfte sowie Erzieher*innen in Kitas zu allgemeiner und IKT-gestützter Elternzusammenarbeit in der Corona-Schließzeit?
  H2: Es wird davon ausgegangen, dass sich Leitungskräfte und Erzieher*innen in ihren Einstellungen bzgl. des Kontakts, bzw. des digital-gestützten Kontakts signifikant unterscheiden.
- Welche Rolle spielen die Qualifikation, das Alter, das Geschlecht und die Position der Fachkräfte sowie ihre Einstellungen im Hinblick auf den Bildungsauftrag und die Elternzusammenarbeit in der Corona-Schließzeit?
  H3: Es wird vermutet, dass die Qualifikation, das Alter, das Geschlecht und die Position der Fachkräfte sowie ihre Einstellungen im Hinblick auf den Bildungsauftrag und die Elternzusammenarbeit signifikant bedeutsam sind.
- Welche Rolle spielen die Qualifikation, das Alter, das Geschlecht und die Position der Fachkräfte, sowie ihre Einstellungen, Erwartungen und ihre wahrgenommene Unterstützung im Hinblick auf die digitale Kontaktaufnahme zu den Eltern in der Corona-Schließzeit?
  H4: Es wird vermutet, dass die Qualifikation, das Alter, das Geschlecht und die Position der Fachkräfte sowie ihre Einstellungen, Erwartungen und ihre wahrgenommene Unterstützung im Hinblick auf die digitale Kontaktaufnahme zu den Eltern statistisch bedeutsam sind.

## Methode

### Design

Die Datenbasis für die Studie liefert eine querschnittliche Onlinebefragung von frühpädagogischen Fachkräften^4^ in Kitas und Kindertagespflegeeinrichtungen, die im Zeitraum vom 09.04.2020 bis 24.05.2020 mithilfe der Plattform SoSci Survey durchgeführt wurde. Die Umfrage fand somit nach der Schließung aller Einrichtungen als bundesweite Maßnahme gegen die Verbreitung des Corona-Virus am 17. März 2020 statt. Abgesehen von der Notbetreuung von Kindern mit Eltern in systemrelevanten Berufen, gab es in dieser Zeit keine Betreuung der Kinder in den Einrichtungen. Die Rekrutierung erfolgte nach dem Gelegenheitsprinzip deutschlandweit über vollständige Kitalisten einzelner Bundesländer (Bayern, Nordrhein-Westfalen, Sachsen-Anhalt, Bremen), Aufrufe zur Teilnahme in Online-Foren, über soziale Medien, Fachkraftportale, Verteiler der Bundes- und Dachverbände, Stiftungen sowie kooperierende Träger. Durch diese Vorgehensweise bei der Stichprobenziehung konnten in kurzer Zeit mit geringem Aufwand viele Fachkräfte erreicht werden. Der Link war öffentlich zugänglich und konnte unter den Fachkräften weiterverbreitet werden. Der Nachteil einer solchen Vorgehensweise liegt darin, dass sich Selektionseffekte nicht ausschließen lassen. Die Fachkräfte wurden zum Umgang mit den Daten umfangreich aufgeklärt und die Teilnahme erfolgte freiwillig. Auch wenn an der Studie Fachkräfte aus verschiedenen Settings der Kindertagesbetreuung teilgenommen haben, bezieht die vorliegende Studie nur die Fachkräfte (Leitungskräfte und Erzieher*innen) in Kitas ein.

### Stichprobe

Die Stichprobe für die vorliegende Studie setzt sich aus 3513 frühpädagogischen Fachkräften in Kitas zusammen. Von diesen waren 24 % (auch) Leitungskräfte, 53 % Erzieher*innen, 3 % Auszubildende sowie 2 % als Springer*in, in Zeitarbeit oder als Praktikant*in tätig. Etwa 18 % der Fachkräfte konnten sich keiner Kategorie zuordnen und haben „Sonstiges“ angegeben. Die Stichprobe verteilt sich über alle Bundesländer, wobei die meisten Fachkräfte in Bayern (19 %), Nordrhein-Westfalen (19 %) und Baden-Württemberg (18 %) tätig waren. In der Notbetreuung waren 37 % der Fachkräfte beschäftigt, 19 % waren zwar in den Einrichtungen, arbeiteten aber nicht mit Kindern, 25 % der Fachkräfte arbeiteten von zu Hause aus während sich 2 % in Kurzarbeit befanden. Weitere 17 % gaben Mischformen an, z. B. Home-Office und Gruppenarbeit wechselten sich ab. Fachkräfte, die keine Erzieher*in oder Leitungskraft waren sowie freigestellt (3 %), beurlaubt (0,5 %) oder arbeitsunfähig (0,5 %) waren, wurden aus allen Angaben und Analysen ausgeschlossen.

### Instrumente

Die frühpädagogischen Fachkräfte wurden zu ihrem Umgang mit der aktuellen Tätigkeitssituation befragt, wie sie die Elternzusammenarbeit (aktuell) gestalten, welche Einstellungen sie zur Nutzung von digitalen Medien für die Zusammenarbeit mit den Eltern haben und wie sie ihre Rolle und Verantwortung als Fachkraft derzeit einschätzen. Im Folgenden werden die für die Untersuchung der Fragestellung verwendeten Konstrukte beschrieben.

#### Kontakt

Die Fachkräfte wurden gefragt, ob sie Kontakt zu den Familien haben (ja/nein) und wer den Kontakt initiiert hat (Eltern/Kita). Ebenfalls wurden sie gefragt, ob sie die Eltern regelmäßig kontaktieren und z. B. Tipps zu regelmäßigen Aktivitäten geben (4-stufig, 1 = stimme gar nicht zu, 4 = stimme voll zu). Weiterhin haben die Fachkräfte angegeben, ob der Kontakt über IKT stattfindet oder nicht, und wenn ja, welche IKT zur Kontaktaufnahme genutzt wurden.

#### Einstellungen

Die Einstellungen, die in dieser Studie erfasst wurden, umfassen sowohl die Einstellungen der Fachkräfte zu IKT als auch zur Elternzusammenarbeit. Die Fachkräfte wurden auf einer 7‑stufigen Skala gefragt, wie wichtig sie den Bildungs‑, Erziehungs- und Betreuungsauftrag von Einrichtungen einschätzen, jeweils im Vergleich zu vor der Pandemie (1 = viel unwichtiger, 7 = viel wichtiger). Dieser Frage liegt die Annahme zugrunde, dass die wahrgenommene Bedeutung des Bildungsauftrags bedeutsam dafür ist, inwiefern der Kontakt mit den Eltern aufgenommen wird. Die Rolle der Fachkraft bezüglich der Zusammenarbeit mit den Familien wurde mithilfe von 5 Items auf einer 4‑stufigen Skala (1 = stimme gar nicht zu, 4 = stimme vollkommen zu) erfasst (Beispielitem: „Gerade in der Zeit der Kita-Schließungen fühle ich mich verpflichtet, die Eltern bei der Förderung der Kinder zu unterstützen, z. B. durch die Bereitstellung von Materialien.“, Cronbachs alpha = 0,76, Eigenentwicklung). Mit einem 10-stufigen Item wurden die Fachkräfte gefragt, wie ihre Einstellungen zum Einsatz digitaler Medien in der Elternzusammenarbeit sind (1 = sehr negativ, 10 = sehr positiv) und ob sich dies im Vergleich zur Zeit vor der Pandemie geändert hat (3-stufig, 1 = negativer als vor der Schließzeit, 3 = positiver als vor der Schließzeit). Die Reaktion, die Fachkräfte von den Eltern erwarten bzw. erwarten würden, wenn sie mithilfe von IKT Kontakt aufnehmen würden, wurde über eine 4‑stufige Skala (1 = stimme gar nicht zu, 4 = stimme vollkommen zu) mit 8 Items abgefragt (Beispielitem: „Eltern reagieren befürwortend“, Cronbachs alpha = 0,87, Eigenentwicklung). Fachkräfte, die angaben, keinen digitalen Kontakt zu den Familien zu haben, berichteten zusätzlich die Gründe, die sie vom IKT-Einsatz in der Kontaktaufnahme zu den Eltern abhalten. Eine Auswahl von 10 Items beinhaltete sowohl Aspekte der technischen Ausstattung, der datenschutzrechtlichen Bedenken und der eigenen Kompetenzen. Mehrfachangaben waren möglich.

#### Strukturmerkmale

Als Strukturmerkmal auf Einrichtungsebene wurde die Einrichtungsgröße erfasst, die über die Anzahl der regulär angemeldeten Kinder pro Einrichtung definiert wurde.

#### Fachkraftmerkmale

Merkmale auf Fachkraftebene sind das Geschlecht (weiblich = 1), das Alter (in Jahren) und die Qualifikation der Fachkraft. Diese wurde zum einen über das Qualifikationslevel (Hochschulabschluss = 1, Berufsausbildungsabschluss = 0) und zum anderen über den frühpädagogischen Schwerpunkt in der Ausbildung (=1) definiert, unabhängig davon, ob ein Studium oder eine Berufsausbildung absolviert wurde. Ebenfalls wurde unterschieden, ob eine Fachkraft Erzieher*in ist oder eine Leitungsfunktion in der Einrichtung hat (Leitung = 1).

Die Verteilung der Merkmale (vgl. Tab. 1) deutet darauf hin, dass die Stichprobe im Hinblick auf die Qualifikation verzerrt ist. Bei den Leitungskräften haben 41 % einen Hochschulabschluss, bei den Fachkräften sind es 21 %. Beide Anteile liegen deutlich über dem regulären Anteil von Hochschulabschlüssen in frühpädagogischen Bildungseinrichtungen in den verschiedenen Bundesländern (Bundesdurchschnitt 6 % (Autorengruppe Bildungsberichterstattung 2020, S. 93)).

**Table 1:** 

**Strukturmerkmale**	***n***	**%**	**M (SD)**
*Anzahl der angemeldeten Kinder*	2842	–	82,13 (49,42)
**Fachkraftmerkmale**			
*Stellung in der Kita*	2406	–	–
Erzieher*in	–	68,66	–
Leitung	–	31,34	–
*Art der Tätigkeit*	3053	–	–
% Notbetreuung	–	36,62	–
% keine Notbetreuung	–	63,38	–
*Qualifikationslevel*	3460	–	–
% Hochschulabschluss	–	29,62	–
*Schwerpunkt*	3205	–	–
% Frühpädagogik	–	87,64	–
*Alter*	3333	–	39,53 (11,57)
*Geschlecht weiblich*	3363	93,90	–

#### Unterstützung

Die durch die Fachkräfte wahrgenommene Unterstützung bei der Implementierung von IKT zur Elternzusammenarbeit wurde mittels zweier Items abgefragt, die die technische und die pädagogische Unterstützung unterscheiden (Item: „Ich erhalte in meiner Einrichtung bei Bedarf technische Unterstützung“; Item: „Ich erhalte in meiner Einrichtung bei Bedarf pädagogische Unterstützung“; 4‑stufig: 1 = sehr wenig, 4 = sehr viel). Zusätzlich wurden die Fachkräfte danach gefragt, inwiefern sie den Träger, das eigene Team, externe Personen, Multiplikator*innen oder niemanden als unterstützend in der Anwendung von IKT in der Elternzusammenarbeit wahrnehmen (Dummyvariablen).

### Analyseverfahren

Zur Beantwortung der Frage nach der Gestaltung der Elternzusammenarbeit und den Einstellungen der Fachkräfte zu IKT-gestützter Elternzusammenarbeit in der Corona-Schließzeit, werden die Angaben der Fachkräfte deskriptiv beschrieben. Unterschiede zwischen Leitungskräften und Erzieher*innen werden mithilfe von T‑Tests bzw. Chi^2^-Tests überprüft. Fachkräfte, die in der Notbetreuung tätig waren, werden aus den deskriptiven Analysen ausgeschlossen, da diese weiterhin einen persönlichen Kontakt zu den Eltern pflegen konnten.

Um die Frage nach den Prädiktoren für eine (digitale) Kontaktaufnahme der Einrichtungen zu den Familien zu beantworten, werden logistische Regressionen durchgeführt und die Ergebnisse als Odds Ratios (OR) präsentiert. Für die abhängige Variable „Kontakt zu den Familien“ werden zwei Modelle berichtet. Im ersten Modell (a) sind ausschließlich die strukturellen Merkmale der Einrichtung und der Fachkraft enthalten. Im zweiten Modell (b) werden die Einstellungsmerkmale der Fachkraft zusätzlich in das Modell aufgenommen. Für das Modell „digitaler Kontakt“ wird ein drittes Modell (c) geschätzt, welches zusätzlich die Unterstützungsmerkmale für den IKT-Einsatz einbezieht. In allen Modellen wird für die Tätigkeit in der Notbetreuung kontrolliert.

Fehlende Werte werden mit dem Full-Information-Maximum-Likelihood-Verfahren (FIML) behandelt (Lüdtke et al. 2007). Die Analysen wurden mit Stata SE 16 (2019) und Mplus 8.4 (Muthén und Muthén 1998–2019) durchgeführt.

## Ergebnisse

### Deskriptive Ergebnisse

Etwa 82 % der Fachkräfte geben an, Kontakt zu den Familien zu haben. Leitungskräfte (96 %) berichteten dies signifikant häufiger (*x*^*2*^ (1, *N* = 1266) = 83,84, *p* = 0,00) als Erzieher*innen (76 %). Der Kontakt ging dabei in der Mehrzahl von den Fachkräften aus (95 %). Auch wenn fast alle Einrichtungen Kontakt zu den Eltern hatten, stimmte nur ein Drittel der Fachkräfte der Aussage vollkommen zu, regelmäßig Kontakt zu den Familien zu haben und z. B. Tipps für Aktivitäten zu geben.

Im Hinblick auf die aktuelle Wichtigkeit des Betreuungs‑, Bildungs-, und Erziehungsauftrages von Kindertageseinrichtungen im Vergleich zu der Zeit vor der Corona-Pandemie schätzen Fachkräfte alle drei Komponenten als eher gleich wichtig ein, den Erziehungsauftrag jedoch am wenigsten (*M* = 4,06, *SD* = 1,68). Es lassen sich diesbezüglich keine signifikanten Unterschiede zwischen der Einschätzung der Erzieher*innen und der Leitungskräfte nachweisen. Mit Blick auf die wahrgenommene Rolle in Bezug auf die Unterstützung der Familien zeigte sich, dass Leitungskräfte (*M* = 3,31, *SD* = 0,57) ihre Rolle im Vergleich zu den Erzieher*innen (*M* = 2,99, *SD* = 0,65) als signifikant bedeutsamer einschätzten (*t*(1292) = −8,98, *p* = 0,00).

In Bezug auf die IKT-gestützte Elternzusammenarbeit gaben etwa 75 % der Fachkräfte an, IKT zur Kontaktaufnahme einzusetzen. E‑Mails wurden mit 76 % am häufigsten genutzt, Messenger (28 %) und geschützte Online-Portale (25 %) seltener. Mehr als zwei Drittel der Fachkräfte (70 %) gaben an, in der Corona-Schließzeit häufiger IKT in der Elternzusammenarbeit einzusetzen als vor der Corona-Schließung. Leitungskräfte (*M* = 8,41, *SD* = 1,76) berichten signifikant positivere Einstellungen (*t*(1207) = −6,12, *p* = 0,00) gegenüber IKT in der Elternzusammenarbeit als Erzieher*innen (*M* = 7,70, *SD* = 2,08). Gleichzeitig gaben 44 % der Fachkräfte, sowohl Leitungskräfte als auch Erzieher*innen, an, insgesamt positiver gegenüber IKT in der Elternzusammenarbeit eingestellt zu sein als noch vor der Pandemie.

Die wahrgenommene bzw. angenommene Reaktion der Eltern auf eine IKT-gestützte Kontaktaufnahme durch die Kitas wurde als positiv berichtet (*M* = 3,18, *SD* = 0,52). Es zeigte sich dabei kein Unterschied zwischen Leitungen und Erzieher*innen. Jedoch nahmen Fachkräfte, die Kontakt über IKT hatten (*M* = 3,28, *SD* = 0,50), eine signifikant positivere Reaktion der Eltern an (*t*(1288) = −12,93, *p* = 0,00) als Fachkräfte, die keinen digitalen Kontakt zu den Familien aufgenommen hatten (*M* = 2,87, *SD* = 0,45). Letztere wurden ebenfalls nach den Hinderungsgründen gefragt, IKT für die Elternzusammenarbeit einzusetzen. Am häufigsten gaben die Fachkräfte datenschutzrechtliche Bedenken an (*M* = 2,92, *SD* = 0,87), gefolgt von unzureichender Hard- und Software (*M* = 2,88, *SD* = 0,93), mangelnder technischer und pädagogischer Unterstützung beim Einsatz (*M* = 2,79, *SD* = 0,86) und mangelnden Schulungen (*M* = 2,63, *SD* = 0,88). Als weniger hinderlich wurden die zeitlichen Ressourcen (*M* = 2,10, *SD* = 0,83) und die eigenen technischen Kenntnisse (*M* = 1,95, *SD* = 0,88) wahrgenommen. Bezüglich dieser Hindernisse gaben Erzieher*innen (*M* = 2,58, *SD* = 0,88) signifikant häufiger als Leitungskräfte (*M* = 2,30, *SD* = 0,82) an, durch Richtlinien der Einrichtung selbst eingeschränkt zu sein (*t*(197) = 2,21, *p* = 0,03).

Insgesamt zeigen die ersten deskriptiven Ergebnisse, dass sich Erzieher*innen und Leitungskräfte nicht prinzipiell in ihren Einstellungen unterscheiden. Dennoch zeigen sich auch differenzielle Ergebnisse für Erzieher*innen und Leitungskräfte, welche jedoch auch durch das unterschiedliche Alter und die unterschiedliche Qualifikation der Fachkräfte bedingt sein können. Zusätzlich kann angenommen werden, dass sich Erzieher*innen und Leitungskräfte in ihrem Tätigkeitsspektrum und Rollenverständnis unterscheiden. Aus diesem Grund wird in den folgenden Analysen die Stellung der Fachkräfte in der Einrichtung weiterhin berücksichtigt.

### Zusammenhang zwischen strukturellen Merkmalen, Einstellungen und der Elternzusammenarbeit

Im Folgenden wird der Frage nachgegangen, welche Rolle strukturelle Merkmale der Einrichtungen, Fachkraftmerkmale sowie die Einstellungen und die wahrgenommene Unterstützung der Fachkräfte im Hinblick auf allgemeine und digitale Elternzusammenarbeit spielen.

Die Ergebnisse der logistischen Regressionen in Tab. 2 zeigen, dass Leitungskräfte und Erzieher*innen ein unterschiedliches Kontaktverhalten zu den Eltern berichteten. Leitungskräfte hatten eine höhere Wahrscheinlichkeit, Kontakt mit den Familien aufzunehmen, als Erzieher*innen. Ebenfalls zeigte sich ein signifikanter Geschlechtereffekt; weibliche Fachkräfte nahmen wahrscheinlicher Kontakt auf als ihre männlichen Kollegen. Unter Berücksichtigung der Einstellungsmerkmale (Modell b) verschwand dieser Effekt jedoch. Dies deutet darauf hin, dass der Einfluss des Geschlechts auf positivere Einstellungen der Erzieherinnen im Vergleich zu den Erziehern zurückzuführen ist. Ein leicht negativer Effekt zeigte sich in beiden Modellen bezüglich des Alters der Fachkräfte: Je jünger die Fachkraft, desto wahrscheinlicher nahm sie Kontakt zu den Familien auf. Im Hinblick auf die Einstellungen zeigte sich, dass die eigene Rollenwahrnehmung in Bezug auf die Elternzusammenarbeit einen sehr großen positiven und signifikanten Effekt auf die Elternzusammenarbeit hatte: Fachkräfte, die für sich reflektierten, dass sie trotz Schließzeit den Kontakt zu den Familien aufrechterhalten müssen, um Familien in der Förderung ihrer Kinder zu unterstützen, nahmen eher Kontakt zu den Familien auf.

**Table 2:** 

	Kontakt	
	(a)	(b)
	OR (SE)	OR (SE)
Einrichtungsmerkmale		
Einrichtungsgröße	0,999 (0,001)	1,000 (0,001)
Fachkraftmerkmale 2		
Stellung (Leitung = 1)	7,723*** (1,617)	6,063** (*1,509*)
Alter	0,987** (0,005)	0,980** (0,006)
Geschlecht (männlich = 1)	0,679* (0,153)	0,982 (0,257)
Qualifikationslevel (Hochschulabschluss = 1)	0,954 (0,128)	*0,904 (0,149)*
Schwerpunkt (Frühpädagogik = 1)	0,891 (0,169)	0,946 (*0,221*)
Einstellungen		
Rolle Elternzusammenarbeit	–	17,393*** (*2,454*)
Wichtigkeit Bildungsauftrag	–	*1,075 (0,072)*
Wichtigkeit Betreuungsauftrag	–	*0,966 (0,045)*
Wichtigkeit Erziehungsauftrag	–	*0,956 (0,074)*

*n* = 3484; kontrolliert für „tätig in Notbetreuung“

*p < 0,05, **p < 0,01 ***p < 0,001

### Zusammenhang zwischen strukturellen Merkmalen, Einstellungen, wahrgenommener Unterstützung und der digitalen Elternzusammenarbeit

Im Hinblick auf den digitalen Kontakt, zeigte Modell a in Tab. 3 zunächst, dass kleinere Einrichtungen signifikant mehr digitalen Kontakt zu den Familien pflegten. Ebenso nutzten jüngere Fachkräfte wahrscheinlicher IKT für die Elternzusammenarbeit. Diese Effekte blieben auch unter Einbezug der Einstellungs- und Unterstützungsmerkmale bestehen. In Modell b wird deutlich, dass die allgemein wahrgenommene Rolle zur Elternzusammenarbeit im Vergleich zu den Modellen zur grundsätzlichen Kontaktaufnahme (Tab. 2) nicht relevant für den digitalen Kontakt war. Demgegenüber zeigte sich in Bezug auf den Einsatz von IKT, dass die Einstellungen zu digitalen Medien eine Rolle spielten. Fachkräfte, die IKT gegenüber positiv eingestellt waren, nutzten IKT auch häufiger für die Elternzusammenarbeit. Weiterhin zeigte sich die von den Fachkräften antizipierte Reaktion der Eltern als bedeutsam. Je positiver die Fachkräfte die Reaktion der Eltern einschätzten, desto wahrscheinlicher setzten sie IKT zur Elternzusammenarbeit ein. In Modell c wurde zusätzlich überprüft, inwiefern die wahrgenommene Unterstützung für den Einsatz in IKT in der Elternzusammenarbeit relevant war. Unter gleichbleibend signifikanter Bedeutsamkeit der Einstellungen zu IKT zeigte sich, dass Fachkräfte wahrscheinlicher IKT einsetzten, wenn sie sich technisch unterstützt fühlten. Die pädagogische Unterstützung spielte dagegen keine Rolle. Zusätzlich war die Unterstützung des Teams und externer Personen relevant für die Umsetzung. Fachkräfte, die vom Team bezüglich des Einsatzes von IKT Unterstützung erfuhren, setzten IKT wahrscheinlicher ein, während eine externe Unterstützung (z. B. durch Institutionen) als hemmend empfunden wurde.

**Table 3:** 

	Digitaler Kontakt		
	(a)	(b)	(c)
	OR (SE)	OR (SE)	OR (SE)
Einrichtungsmerkmale			
Einrichtungsgröße	0,996*** (0,001)	0,995*** (0,001)	0,995*** (0,001)
Fachkraftmerkmale			
Stellung (Leitung = 1)	1,340 (0,183)	*1,193 (0,179)*	*1,309 (0,205)*
Alter	0,979*** (0,005)	0,983** (0,005)	0,985** (0,005)
Geschlecht	1,624 (0,434)	*2,055 (0,600)*	*2,051 (0,609)*
Qualifikationslevel (Hochschulabschluss = 1)	1,191 (0,150)	*1,227 (0,168)*	*1,237 (0,173)*
Schwerpunkt (Frühpädagogik = 1)	1,446 (0,241)	*1,371 (0,249)*	*1,372 (0,255)*
Einstellungen			
Rolle Elternzusammenarbeit	–	*0,911 (0,105)*	*0,870 (0,102)*
Wichtigkeit Bildungsauftrag	–	*1,005 (0,051)*	*0,995 (0,051)*
Wichtigkeit Betreuungsauftrag	–	*0,939 (0,035)*	*0,940 (0,036)*
Wichtigkeit Erziehungsauftrag	–	*1,041 (0,060)*	*1,038 (0,061)*
Reaktion der Eltern	–	2,825*** (0,349)	2,737*** (0,341)
Einstellung zu IKT	–	1,330*** (0,043)	1,310*** (0,043)
Unterstützung			
Technische Unterstützung	–	–	1,248* (0,101)
Pädagogische Unterstützung	–	–	*0,996 (0,082)*
Träger	–	–	*1,039 (0,128)*
Team	–	–	1,493* (0,213)
Extern	–	–	0,601*** (0,082)
Multiplikator*in	–	–	*1,003 (0,194)*
Niemand	–	–	*1,144 (0,258)*

*n* = 3484; kontrolliert für „tätig in Notbetreuung“

*p < 0,05, **p < 0,01 ***p < 0,001

## Diskussion

Im Beitrag wurde der Frage nachgegangen, wie häufig und in welcher Form (digitale) Elternzusammenarbeit in der Corona-Schließzeit von frühpädagogischen Fachkräften stattfand, welche Einstellungen Fachkräfte zur (digitalen) Elternzusammenarbeit in der Schließzeit hatten und welche fachkraftbezogenen und einrichtungsbezogenen Merkmale sowie Überzeugungen in Zusammenhang mit der generellen Kontaktaufnahme zu den Familien und dem Einsatz von IKT in der Elternzusammenarbeit relevant waren.

Die Ergebnisse zeigen, dass der überwiegende Teil der Fachkräfte mindestens einmal Kontakt zu den Familien aufgenommen hat. Deutlich seltener wurde hingegen ein regelmäßiger Kontakt zu den Familien gepflegt und z. B. Fördermöglichkeiten angeboten. Diese Ergebnisse deuten darauf hin, dass die Kitas in der Schließzeit ein Verständnis von Elternzusammenarbeit vertreten hatten, welches über die einmalige Weiterleitung von z. B. Informationen an die Familien selten hinaus ging. Dafür spricht ebenfalls, dass eher Leitungskräfte den Kontakt zu den Familien aufnahmen als Fachkräfte und damit eine weniger individualisierte Kommunikation wahrscheinlicher war. Das muss nicht bedeuten, dass die Erzieher*innen keinen regelmäßigen Kontakt möchten. Wenn man zusätzlich davon ausgeht, dass drei Viertel der Fachkräfte während der Schließzeit IKT für die Kontaktaufnahme genutzt haben, kann man auf Basis bisheriger Studien und der vorliegenden Ergebnisse annehmen, dass auf digitalem Weg vor allem Informationen verbreitet werden und Fachkräfte und Eltern andere Kommunikationsformen für komplexere Themen bevorzugen (Hu et al. 2009; Thompson et al. 2015). Die Präferenz für den persönlichen Austausch wird auch durch die bisherige Umsetzung der Elternzusammenarbeit in den Einrichtungen deutlich. So waren bis vor der Schließzeit tägliche Tür- und Angelgespräche, individuelle Eltern- und Entwicklungsgespräche, Feste und Elternabende in über 90 % der Kitas verbreitet (Viernickel et al. 2013). Es ist zu fragen, inwieweit die bisherigen Angebote in der Schließzeit digital weitergeführt wurden (z. B. Elterngespräche) oder auch Angebote entwickelt wurden, die den Wegfall kompensierten.

Gleichzeitig zeigen die Ergebnisse der vorliegenden Studie, dass Fachkräfte die Wichtigkeit ihrer Rolle in Bezug auf den umzusetzenden Bildungsauftrag in der Elternzusammenarbeit auch in der Corona-Schließzeit ohne den direkten Kontakt mit Kindern und Eltern durchaus wahrnehmen. Die Diskrepanz zwischen der Reflektion dessen und der Umsetzung könnte darauf zurückzuführen sein, dass Einrichtungen keine Konzepte dazu entwickelt hatten, wie sie eine (digitale) Elternzusammenarbeit gestalten können, die sowohl die Eltern darin unterstützt ein optimales Entwicklungs- und Lernumfeld für ihre Kinder zu schaffen als auch den eigenen Bildungsauftrag integriert.

Insgesamt zeigte sich, dass Fachkräfte insbesondere E‑Mails, aber auch Messenger und Portale während der Corona-Schließzeit häufiger nutzten als noch vor der Schließzeit. Der Anstieg in der Nutzung von IKT für die Elternzusammenarbeit zeigt sich besonders im Vergleich zu früheren Forschungsergebnissen. Zum Beispiel berichtete (Knauf 2020b), dass nur ein geringer Anteil der Fachkräfte IKT nutzten (9 % E‑Mail).

Im Hinblick auf den Zusammenhang zwischen den Einstellungen zu IKT und dem tatsächlichen Einsatz konstatieren Kim et al. (2013) und Tondeur et al. (2017), dass die Richtung bisher nicht eindeutig ist, denn Veränderungen der Einstellungen können sowohl Verhaltensänderungen anstoßen als auch auf Verhaltensänderungen beruhen. Die Fachkräfte der Corona-Studie berichteten nach eigener Einschätzung, dass sie im Vergleich zu vor der Schließzeit deutlich positiver gegenüber IKT im Kitakontext eingestellt sind. Bezugnehmend auf die unklare Richtung des Zusammenhangs, lässt sich dies möglichweise dadurch erklären, dass Fachkräfte durch die Umstände während der Pandemie-Schließzeit vermehrt die Notwendigkeit gesehen haben, IKT zu nutzen, auch wenn sie vorher dazu nicht bereit waren oder keine Ressourcen hatten. Der akute Handlungsdruck der Fachkräfte und die persönliche Erfahrung, dass digitale Medien hilfreiche Werkzeuge in der Zusammenarbeit sein können, kann ebenso dazu geführt haben, dass sich Einstellungen diesbezüglich änderten. Knauf (2020b) berichtet jedoch, im Gegensatz zu bisherigen Studien, dass Fachkräfte grundsätzlich positiv gegenüber dem Einsatz von IKT in Kitas eingestellt sind. Die schnellen Veränderungen könnten dennoch auch bei grundsätzlich positiv eingestellten Fachkräften zur Überwindung bisher berichteter Hinderungsgründe und somit zu einer höheren Implementationsquote geführt haben.

Interessanterweise zeigen die Ergebnisse ebenfalls, dass die wahrgenommene Rolle zur Elternzusammenarbeit keinen Einfluss darauf hatte, ob IKT eingesetzt wurde oder nicht. Das bedeutet: *Ob* Fachkräfte überhaupt Kontakt zu den Familien aufnahmen, hing von ihrer wahrgenommenen Wichtigkeit als Fachkraft im Hinblick auf die Unterstützung der Eltern ab. *Wie* dieser Kontakt dann hergestellt wurde, hing wiederum von ihren Einstellungen zu IKT ab. Dieses Befundmuster zeigt sich auch in der internationalen Forschung, wenn es um den allgemeinen pädagogischen Einsatz von IKT geht (Blackwell et al. 2014; Plumb und Kautz 2015). Die Einstellungen zu IKT sind prädiktiv dafür, wie digitale Medien in der pädagogischen Arbeit mit Kindern integriert werden.

Nikolopoulou und Gialamas (2015) und Marklund (2019) konnten in ihren Studien zeigen, dass die Einstellungen der Eltern für den Implementationsprozess von IKT in frühkindlichen Bildungseinrichtungen relevant sind. Die Ergebnisse der vorliegenden Studie bestätigen, dass die antizipierten Einstellungen und Reaktionen der Eltern für die Entscheidung relevant waren, IKT in der Kommunikation mit den Eltern einzusetzen. Die Richtung des Zusammenhangs kann in der vorliegenden Studie jedoch nicht geklärt werden: Kontaktieren Fachkräfte Eltern in digitaler Form vor allem dann, wenn sie wissen, dass Eltern generell positiv darauf reagieren würden? Oder sehen die Fachkräfte die antizipierte negative Reaktion der Eltern als Hindernis oder auch als Grund, um sich nicht mit IKT auseinandersetzen, ohne tatsächlich eine Erfahrung mit der Reaktion der Eltern zu haben? Die antizipierte Reaktion der Eltern könnte aber auch ein Indikator für die generelle Wahrnehmung der Elternschaft und Elternzusammenarbeit durch die Fachkraft sein. Das heißt, Fachkräfte, die die Elternzusammenarbeit und den Kontakt mit den Eltern generell eher als belastend oder negativ sehen, würden auch in der Schließzeit wenig Motivation haben diese aufrecht zu erhalten.

Ebenso zeigten sich unterstützende Maßnahmen als relevant für die IKT-Nutzung in der Elternzusammenarbeit. Dabei war es für Fachkräfte wichtiger eine technische Unterstützung als eine pädagogische Unterstützung bezüglich der Art und Weise der Umsetzung zu erhalten. Dieser nicht signifikante Effekt könnte sich dadurch erklären, dass Fachkräfte bereits mit dem technischen Umgang von IKT herausgefordert waren, ohne darüber zu reflektieren, dass es auch pädagogischer Fähigkeiten bedarf, Elternzusammenarbeit und den Kontakt zu den Kindern über IKT im Hinblick auf den Bildungsauftrag zu gestalten. Wenn man davon ausgeht, dass Fachkräfte vor allem Informationen an Eltern weiterleiteten und keine individualisierten Angebote und Unterstützungsmaßnahmen für Familien und Kinder konzipierten, brauchte es entsprechend auch kein Wissen darüber, *wie* IKT pädagogisch effektiv eingesetzt werden können.

Dennoch sind für die Fachkräfte, so zeigen die Ergebnisse, ebenfalls verschiedene Unterstützungsinstanzen für die IKT-Nutzung relevant. Vor allem die Unterstützung durch das Team hatte einen positiven Effekt auf den Einsatz. Auch dieser Befund bestätigt die Ergebnisse der internationalen Literatur, die zeigt, dass insbesondere die Rolle der Leitung relevant zu sein scheint (Bordalba und Bochaca 2019; Jeong und Kim 2017). Interessanterweise hatte eine Unterstützung durch Externe (z. B. Träger) einen negativen Effekt. Denkbar wäre, dass Fachkräfte durch externe Institutionen einen Anforderungsdruck oder eine Überfrachtung von Informationen wahrnehmen. Es spiegelt sich damit eine typische Logik der Kinder- und Jugendhilfe wider, dass äußere Interventionen oder oktroyierte Maßnahmen als störend oder gar Gefahr empfunden werden. Das Ergebnis könnte aber auch darauf hindeuten, dass jede Einrichtung für sich individualisierte Strategien für eine umfassende Implementation von IKT gemeinsam mit dem Team entwickeln muss und dass bisherige Professionalisierungsangebote zu sehr darauf abzielen, einzelne Fachkräfte fortzubilden, die die Mediatisierung der Kitas vorantreiben sollen (Knauf 2020a).

Interessant ist ebenfalls der negative Alterseffekt in der Umsetzung von IKT. Während Friedrichs-Liesenkötter (2016b) und auch Knauf (2020b) keine stärkere Offenheit gegenüber IKT in der pädagogischen Praxis bei jüngeren im Vergleich zu älteren Fachkräften feststellte, zeigte sich in der vorliegenden Studie ein negativer Effekt. Der negative Effekt könnte aber erst durch die konkrete Notwendigkeit des Einsatzes entstehen, während die Einstellungen im Hinblick auf einen möglichen Einsatz in den Studien von Friedrichs-Liesenkötter (2016b) und Knauf (2020b) altersneutral sind. Ebenso kann angenommen werden, dass sich hier unterschiedliche Zusammenhänge in der Elternzusammenarbeit im Vergleich zum Einsatz von IKT in der pädagogischen Arbeit mit Kindern zeigen.

Fachkräfte berichten eine Reihe von Gründen, die sie an der Umsetzung von IKT-gestützter Elternzusammenarbeit nach hindern. Die mangelnde technische Ausstattung und Unterstützung bei der Umsetzung wurde auch in anderen Studien genannt (Plumb und Kautz 2015). Ein bisher in der internationalen Literatur weniger bekanntes Thema sind datenschutzrechtliche Bedenken der Fachkräfte (Plumb und Kautz 2015). Dass Fachkräfte diesen Aspekt in der vorliegenden Studie als häufigste Barriere angeben, mag zum einen daran liegen, dass im deutschen Kontext ein sehr starkes Bewusstsein datenschutzrechtlicher Bestimmungen existiert und diesem durch die Einführung der DSGVO nochmals öffentliche Aufmerksamkeit zuteilwurde. Zum anderen zeigt es, dass hier ein dringender Klärungsbedarf auf Steuerungsebene und bei den Fachkräften herrscht, insbesondere beim Einsatz digitaler Medien im Kontext von Kitas und Home-Office-Tätigkeiten. Zeitliche Ressourcen wurden in der vorliegenden Studie weniger stark als Barriere wahrgenommen als in bisherigen Studien (Six et al. 2007). Das liegt sicher zum einen an der veränderten Arbeitstätigkeit während der Corona-Schließzeit. Zum anderen kann es aber auch ein Hinweis darauf sein, dass Fachkräfte, insbesondere technisch versiertere, zunehmend die Erfahrung machen, dass IKT den zeitlichen Aufwand von Tätigkeiten reduzieren können (Piper et al. 2013).

Die eingangs gestellte Frage ob in Kitas während der Corona-Pandemie ein Digitalisierungsschub stattgefunden hat und ob frühpädagogische Fachkräften die Chance genutzt haben, um über digitale Wege mit Familien in Kontakt zu treten, Beziehungen aufrecht zu erhalten und ihrem Bildungsauftrag nachzukommen, muss mit Jein beantwortet werden. Es ist möglich, dass die schnelle und sehr starke Veränderung des pädagogischen Alltags von Fachkräften die Einstellungen zu digitalen Medien grundsätzlich und insbesondere in der Zusammenarbeit mit Eltern positiv beeinflusst hat und in diesem Sinne einen „Digitalisierungsschub“ angestoßen hat. Auf der anderen Seite wird deutlich, dass der Einsatz von IKT immer noch nicht konzeptionell verankert ist und vornehmlich nur für eine Form der Zusammenarbeit genutzt wird, nämlich für die Verbreitung von Informationen. Die Vielfalt der Möglichkeiten und das volle Potenzial von IKT, sowohl in der Elternzusammenarbeit als auch in einer qualitativ hochwertigen Umsetzung des Bildungsauftrages in der Zusammenarbeit mit den Familien, wurden von der Mehrheit der Befragten nicht genutzt.

### Limitationen

Die Ergebnisse der Studie müssen vor dem Hintergrund folgender Limitierungen interpretiert werden. Zum einen handelt es sich um Querschnittsdaten, die Entwicklungen vor, während der Corona-Schließzeit oder auch langfristige Effekte nicht abbilden können. Somit ist es nicht möglich zu klären, ob die positiven Einstellungen Voraussetzung oder Konsequenz des IKT-Einsatzes sind. Weiterhin macht das hohe Qualifikationslevel der Befragten deutlich, dass es sich um eine selektive Stichprobe von Fachkräften handelt, die sich auch in anderen Merkmalen von einer repräsentativen Stichprobe unterscheiden kann, z. B. hinsichtlich der Einstellungen und Erfahrungen mit dem IKT-Einsatz oder auch dem Vorhandensein digitaler Infrastrukturen in den Einrichtungen. Diese Selektionseffekte können ebenfalls dafür verantwortlich sein, dass die Qualifikation der Fachkräfte in den Ergebnissen nicht zum Tragen kommt. Das Online-Befragungsformat sowie eine Rekrutierungsstrategie, die zum einen nach dem Gelegenheitsprinzip und zum anderen weitestgehend über digitale Kanäle stattfand, könnten eine Ursache für eine solche Selektion sein.

Ebenfalls muss limitierend angefügt werden, dass die Erfassung der Einstellungen in der Studie sehr begrenzt war. Hier ist es für weitere Untersuchungen unabdingbar, die theoretische Breite des Konstrukts auch empirisch zu erfassen (z. B. motivationale Aspekte, Einstellungen zu bestimmten Aspekten von Elternzusammenarbeit). Ebenfalls kann die eher allgemeine Frage danach, ob ein Kontakt zu den Eltern stattgefunden hat, nicht umfassend und detailliert genug beantwortet werden, denn ob eine Fachkraft zu einem Elternteil Kontakt hatte oder zu mehreren Eltern oder ob eine gruppenbasierte bzw. individuelle Kontaktaufnahme erfolgte, kann nicht nachvollzogen werden. Ebenfalls muss bedacht werden, dass die Position der Leitungskraft bedeuten kann, dass sie vom Gruppendienst freigestellt oder auch im Gruppendienst tätig ist. Die Fragen könnte eine Leitungskraft, wenn sie nicht freigestellt ist, sowohl in ihrer Rolle als Leitungskraft als auch als Fachkraft im Gruppendienst beantwortet haben. Für die Studie wurden von den Fachkräften keine Informationen zur technischen Ausstattung der Einrichtung erhoben, welche sich bereits in anderen Studien als relevant für die Nutzung von IKT gezeigt haben. Es wurde darauf verzichtet, da die Art der aktuellen Tätigkeit und der Arbeitsort unklar waren (z. B. Tätigkeit im Home-Office). Eine eindeutige Rückführung auf die Ausstattung der Einrichtung wäre somit nicht möglich gewesen. Es ist davon auszugehen, dass eine Reihe von Fachkräften aufgrund der Dringlichkeit und der unzureichenden Ausstattung der Einrichtungen auf private Geräte zurückgriff.

### Implikationen

Die Ergebnisse zeigen, dass insbesondere die Einstellungen der Fachkräfte gegenüber IKT sowie die Unterstützung die Nutzung von IKT zur Elternzusammenarbeit während der Corona-Schließzeit begünstigt. Weitere Forschung ist notwendig, die nicht nur die Quantität des Kontakts, sondern auch die Qualität der digitalen Zusammenarbeit untersucht, auch um herauszufinden, inwiefern die Nutzung digitaler Formate zur Elternzusammenarbeit auch nachhaltig in den Einrichtungen implementiert bleiben kann. Ebenfalls sollte in zukünftigen Untersuchungen und in der Praxis die Perspektive der Eltern stärker einbezogen werden. Es kann nicht davon ausgegangen werden, dass alle Eltern den Wunsch, die Ausstattung, einen Zugriff und die technologischen Kompetenzen haben IKT in der Elternzusammenarbeit vollständig und auch langfristig anzunehmen. Dabei soll noch einmal betont werden, dass IKT in einem Regelbetrieb der Einrichtungen den persönlichen face-to-face Kontakt nicht ersetzen sollen, sondern ein zusätzliches Angebot darstellen, um Eltern zu erreichen, die das herkömmliche Angebot aus verschiedenen Gründen nicht wahrnehmen können. Hinzu kommt, dass es auch im Regelbetrieb für die meisten Eltern bereits vor der Corona-Pandemie Realität war, die Bezugserzieher*in nicht persönlich anzutreffen, und umgekehrt. So forderte erst jüngst die Autorengruppe Bildungsberichterstattung (2020) zeit- und ortsunabhängige personalisierte Angebote durch Bildungseinrichtungen zu schaffen, um damit den pädagogischen Handlungsspielraum zu erweitern und die Teilhabe aller Eltern und Kinder an Bildungsgelegenheiten zu sichern. In diesem Sinne können und sollten frühkindliche Bildungseinrichtungen aus den Erfahrungen der coronabedingten Schießzeit lernen und nachhaltige Digitalisierungsprozesse in Kitas in Gang setzen, die digitalisierte Elternzusammenarbeit langfristig ermöglichen. Den Ausbildungs- und Fortbildungssystemen kommt eine besondere Bedeutung zu, da sie sowohl die Entwicklung positiver Haltungen und Einstellungen zur digitalen Elternzusammenarbeit begleiten als auch Umsetzungsbeispiele anbieten können. Bei der Implementation von IKT bedarf es auch einer Unterstützung der Einrichtungen dahingehend, Konzepte und Ziele des Einsatzes digitaler Medien zu entwickeln sowie Strategien der Umsetzung zu erproben. Die Fachkräfte müssen schließlich so in ihren professionellen Kompetenzen aufgestellt sein, dass sie einschätzen können, wann und wie ein Einsatz von IKT sinnvoll sein kann und wann nicht. Sowohl Politik, Vertreter auf Steuerungsebene als auch Träger sind gefordert Bedingungen zu schaffen, die diese Änderungen unterstützen.

## Footnotes

1 Informations- und Kommunikationstechnologien (IKT) meint digitale Geräte (Desktops, Laptops, Tablet Computer, Smartphones) und deren Produkte (Apps, Webseiten etc.).

2 Anders, Y., Ballaschk, I., Dietrichkeit, T., Flöter, M., Groeneveld, I., Lee, H.-J., Nattefort, R., Roßbach, H.-G., Schmerse, D., Sechtig, J., Tietze, W., Tuffentsammer, M., Turani, D., Weigel, S., & Wieduwilt, N. (2016). Implementation und Auswirkungen des Bundesprogramms „Schwerpunkt-Kitas Sprache & Integration“: Studie und Ergebnisse im Detail. In H.-G. Roßbach, Y. Anders & W. Tietze (Hg.), Wissenschaftliche Evaluation des Bundesprogramms „Schwerpunkt-Kitas Sprache & Integration“ (S. 22–280, unveröffentlichter Bericht, Freie Universität Berlin, Departement of Early Childhood Education).

3 Anders, Y., Ballaschk, I., Dietrichkeit, T., Flöter, M., Groeneveld, I., Lee, H.-J., Nattefort, R., Roßbach, H.-G., Schmerse, D., Sechtig, J., Tietze, W., Tuffentsammer, M., Turani, D., Weigel, S., & Wieduwilt, N. (2016). Implementation und Auswirkungen des Bundesprogramms „Schwerpunkt-Kitas Sprache & Integration“: Studie und Ergebnisse im Detail. In H.-G. Roßbach, Y. Anders & W. Tietze (Hg.), Wissenschaftliche Evaluation des Bundesprogramms „Schwerpunkt-Kitas Sprache & Integration“ (S. 22–280, unveröffentlichter Bericht, Freie Universität Berlin, Departement of Early Childhood Education).

4 Alle in der Einrichtung unmittelbar oder mittelbar pädagogisch tätigen Personen werden im vorliegenden Beitrag als frühpädagogische Fachkräfte bezeichnet. Als Erzieher*in werden im vorliegenden Beitrag Fachkräfte bezeichnet, die in der Gruppenarbeit pädagogisch tätig sind und sich damit von den Leitungskräften abgrenzen. Fachkräfte mit anderen Abschlüssen (z. B. Kindheitspädagog*in, Sozialassistent*in) sind eingeschlossen.
